# Unveiling Polymerization Mechanism in pH‐regulated Supramolecular Fibers in Aqueous Media

**DOI:** 10.1002/chem.202101660

**Published:** 2021-06-29

**Authors:** Nicolás M. Casellas, Lorenzo Albertazzi, Sílvia Pujals, Tomás Torres, Miguel García‐Iglesias

**Affiliations:** ^1^ Department of Organic Chemistry Universidad Autónoma de Madrid (UAM) Institute for Advanced Research in Chemical Sciences (IAdChem) Calle Francisco Tomás y Valiente, 7 28049 Madrid Spain; ^2^ IMDEA Nanociencia c/ Faraday 9 Cantoblanco 28049 Spain; ^3^ Nanoscopy for Nanomedicine group Institute for Bioengineering of Catalonia (IBEC) The Barcelona Institute of Science and Technology (BIST) Carrer Baldiri Reixac 15–21 08024 Barcelona Spain; ^4^ Department of Biomedical Engineering and ICMS Eindhoven University of Technology 5600 MB Eindhoven The Netherlands; ^5^ Department of Electronics and Biomedical Engineering Faculty of Physics Universitat de Barcelona Av. Diagonal 647 08028 Barcelona Spain; ^6^ QUIPRE Department University of Cantabria Avd. de Los Castros, 46 39005 Santander Spain

**Keywords:** pH responsivity, polymerization mechanism, self-assembly, supramolecular chemistry, supramolecular polymers

## Abstract

An amine functionalized C_3_‐symmetric benzotrithiophene (BTT) monomer has been designed and synthetized in order to form pH responsive one‐dimensional supramolecular polymers in aqueous media. While most of the reported studies looked at the effect of pH on the size of the aggregates, herein, a detailed mechanistic study is reported, carried out upon modifying the pH to trigger the formation of positively charged ammonium groups. A dramatic and reversible change in the polymerization mechanism and size of the supramolecular fibers is observed and ascribed to the combination of Coulombic repulsive forces and higher monomer solubility. Furthermore, the induced frustrated growth of the fibers is further employed to finely control the one‐dimensional supramolecular polymerisation and copolymerization processes.

## Graphical abstract

**The synthesis** and self‐assembly in aqueous media of an amine functionalized C_3_‐symmetric benzotrithiophene is reported. A dramatic change in the polymerization mechanism and size of the supramolecular fibers is observed upon modifying the pH, which is further employed to finely control the one‐dimensional supramolecular polymerization and copolymerization processes.

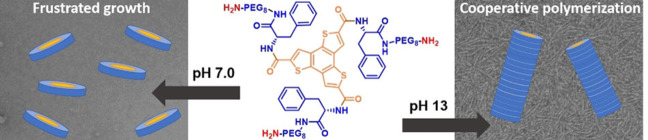

Self‐assembly is a powerful tool to access a great variety of structures, where the reversibility of the supramolecular interactions endows such materials with the possibility to respond towards different external stimuli, a key feature for a plethora of applications.^[1]^

In this context, the rational design of responsive supramolecular 1‐dimensional polymers in water^[2]^ presenting a precise control over shape, size, and stability of the aggregates is of special interest for applications in biomedical technologies.^[3]^ It is, therefore, of paramount importance to find a balance between the different supramolecular interactions^[4]^ to control the polymerization mechanism and the final properties of the aggregates.^[5]^

Several pH‐responsive supramolecular monomers have been described in the last decade, where their supramolecular polymerization can be tuned via electrostatic repulsive forces, varying pH,^[6]^ leading to highly promising materials for biomedical applications, such as pH‐triggered drug delivery.^[7]^

Additionally, the frustrated growth induced by the presence of Coulombic repulsive forces of charged monomeric species have been recently employed to control the shape and size of the resulting supramolecular aggregates with high precision.^[8]^ In this framework, the pioneering work of Besenius et al., unveiled the importance of the polymerization mechanism under the influence of repulsive interactions, and more specifically, the cooperativity as a key property to rule the physical properties of the supramolecular polymers.^[9]^

However, most of the studies reported examples in this area are focused on the size control of the supramolecular polymers or copolymers, while the influence of the Coulombic repulsion forces on the polymerization mechanism and therefore, the structure‐mechanism relations,^[10]^ is generally overlooked.

Herein, we present the design, synthesis and self‐assembly study of a C_3_‐symmetric benzotrithiophene (BTT)^[11]^ monomeric unit where peripheral amine groups have been incorporated in order to introduce electrostatic repulsive forces triggered by pH changes. The detailed experimental study presented unravels key mechanistic aspects of the supramolecular polymerization in the presence of Coulombic interactions. Moreover, this study provides a rationale for the designed control over the size of the supramolecular polymers in aqueous media upon modifying the pH.

The molecular design of the self‐assembling unit **BTT**‐**F**‐**NH_2_**, has been planned based on a monomeric structure recently reported that directs the supramolecular polymerization into strictly one‐dimensional aggregates in a highly cooperative fashion in water (**BTT**‐**F**‐**OH**), providing a good balance between directional hydrogen‐bonding and non‐directional hydrophobic interactions.^[12]^ Terminal amine groups have been incorporated in such structure to the water solubilizing octaethyleneglycol side‐chains, located next to the L‐phenylalanine, in order to confer to the resulting fibrillar structures pH‐responsiveness (see Figure 1a) (see Supporting Information for synthetic characterization).

**Figure 1:**
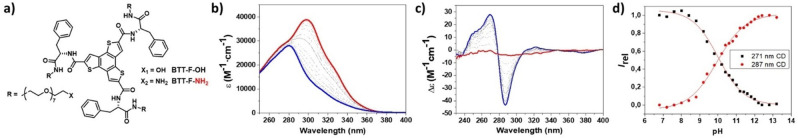
Chemical structures of BTT derivatives. (a). Changes of **BTT**‐**F**‐**NH_2_** ammonium formate (c=5×10^−5^ M) in water at room temperature in (b) absorption and (c) CD intensity as a function of increasing pH by addition of NaOH from 6.8 (red line) to 13.4 (blue line), as a result of the acid‐base equilibrium of terminal amine groups. (d) Normalized CD signal intensity I_rel=_CDx‐CD_min_/CD_max_‐CD_min_ at λ=287 nm (red dots) and λ=271 nm (black dots) of **BTT**‐**F**‐**NH_2_** fitted with the non‐protonated/protonated ratio using the Henderson‐Hasselbalch equation (line) as a function of pH.

Changes in structure of **BTT**‐**F**‐**NH_2_** were monitored as a function of pH by different techniques such as UV‐vis, circular dichroism (CD) spectroscopy and TEM.

The UV spectrum of **BTT**‐**F**‐**NH_2_** ammonium formate at pH 6.8 shows an absorption maximum at 301 nm (Figure 1b, red line), indicating that monomers are molecularly dissolved as a consequence of the high electrostatic repulsion forces between the three‐fold ammonium moieties.^[12]^ In contrast, when increasing pH by stepwise addition of NaOH to the aqueous solution up to pH=13.4, the compound spectrum evolves to an absorption band less intense and hypsochromically shifted, located at 284 nm. This fact indicates the formation of H‐aggregates of BTT cores when the amine groups are in their neutral state (see Figure 1b, blue line). Similar hints were perceived by CD spectroscopy during the pH‐dependent experiments in water, where at pH 6.8 the CD signal is minimal, indicating non‐aggregation between molecules, while a strong bisignated Cotton effect in CD spectra turns up when raising pH (see Figure 1c). This ratifies the formation of helical columnar aggregates with a preferred handedness at elevated pH when the monomers are in their non‐protonated state.). These results are in good agreement with the data previously reported by us showing the characteristic features of BTT derivatives either molecularly dissolved or aggregated in fibrillar structures by UV and CD spectroscopy.[^12^, ^13^]

Moreover, the normalized CD (Figures. 1d and S1) and UV (Figure S2) signals during the pH‐dependent experiments are in perfect agreement with the protonation states and pKa values of the terminal amine groups^[14]^ and can be nicely fitted by the Hendersen‐Hasselbalch equation. This fact points out that along the pH‐dependent experiments, **BTT**‐**F**‐**NH_2_** self‐assembly is triggered by the disappearance of ionic repulsion between the positively charged ammonium groups and the decrease in the monomer solubility. However, the absence of proximity effects lowering the pKa values indicates large distances between ammonium moieties.^[15]^ This fact might indicate the participation of cation‐π interactions between ammonium cations and the electron‐rich aromatic core in the equilibrium, protecting against stacking and deprotonation.^[16]^

The spectroscopic observations were corroborated by TEM negative staining, observing the formation of fibrillar assemblies with a diameter of 5 nm in water at elevated pH, while non‐appreciable objects could be observed at pH 6.5 (see Figure 2).

**Figure 2:**
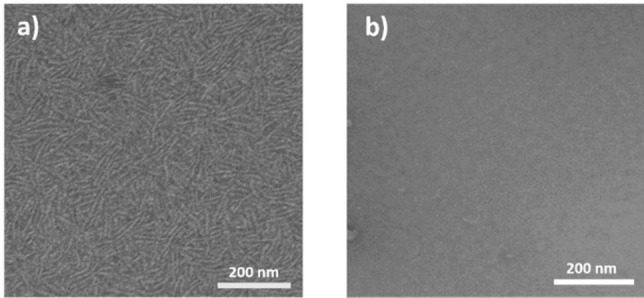
TEM images of **BTT**‐**F**‐**NH_2_** in water (c=4×10^−5^ M) (a) one‐dimensional fibers at pH 13 and (b) non‐appreciable objects at pH 6.5.

In summary, we have demonstrated that the self‐assembly of **BTT**‐**F**‐**NH_2_** into one‐dimensional supramolecular polymers in water can be switched ‘on’ and ‘off’ as a function of pH when introducing Coulombic forces. Moreover, this process is fully reversible (Figure S3).

Once determined the spectroscopic properties and the morphology of this pH‐switchable system, we aim to investigate the interactions driving the self‐assembly under pH influence. Therefore, in order to study the polymerization mechanism of this system, temperature dependent experiments in water were carried out from 280 to 356 K, monitoring changes to the CD, or UV spectra at pH=13 and pH=7 at different monomer concentrations.

According to the temperature dependent experiments at pH 13, a complete disruption of the **BTT**‐**F**‐**NH_2_** supramolecular polymers was observed when raising the temperature (see Figure S4). The different melting curves obtained by CD at different concentrations showed a non‐sigmoidal behavior, suggesting a nucleation‐elongation polymerization process. This fact was confirmed by successfully fitting the data with the cooperative model developed by Eikelder, Markvoort, Meijer and co‐workers^[17]^ (see Figure 3a and S5). Thermodynamic parameters derived from the fitting disclosed a high degree of cooperativity and thermodynamic parameters similar than those previously reported by us for similar structures terminated by ‐OH instead ‐NH_2_ forming highly ordered one‐dimensional supramolecular polymers 12 (see Table 1).

**Figure 3:**
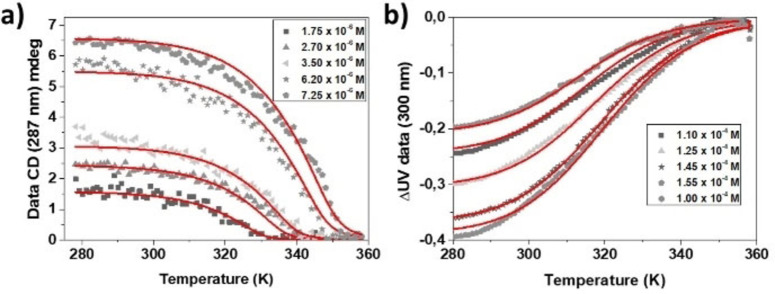
Temperature‐dependent aggregation behavior of. **BTT**‐**F**‐**NH_2_**. Plots of the CD intensity recorded at 287 nm as a function of temperature at different concentrations at pH=13 (a). Plot of the variation of the absorbance of **BTT**‐**F**‐**NH_2_** ammonium formate at λ=300 nm versus temperature at different concentrations (b). The cooling rate for both plots was 2 K min^−1^. The red lines in (a) and (b) correspond to the fit using Eikelder‐Markvoort‐Meijer model.^[17]^

**Table 1:** Thermodynamic parameters obtained from temperature‐dependent CD experiments of **BTT**‐**F**‐**NH_2_** in water at pH 13 at different concentrations on the basis of the ten Eikelder‐Markvoort‐Meijer model^17^. λ=287 nm.

ΔH_ELO_^[a]^ [kJ mol^−1^]	Δ*S* ^[b]^ [Jmol^−1^K^−1^]	Δ*H*_NP_^[c]^ [kJ mol^−1^]	*K*_e_^[d]^* [10^6^ M^−1^]	*K*_n_^[e]^* [M^−1^]	σ^[f]^* [10^−5^]
−65	−85	−26	9.1	214	2.3

[a] Elongation enthalpy. [b] Entropy of elongation. [c] Nucleation penalty. [d] Elongation constant. [e] Nucleation constant. [f] Degree of cooperativity of cooperativity associated with the polymerization process, calculated as *σ*=*K*_n_/*K*_e_. *K_e_, *K*_n_ and σ were calculated at 298 K. The cooling rate was 2 K min^−1^.

Analogous experiments were performed at pH 7, where the presence of aggregated species was only observed when using 10^2^‐fold higher concentrations than the previous experiments carried out at elevated pH, revealing lower stability of the system either by inferior stability of the supramolecular polymer or more favourable interactions between monomer and solvent. At those concentrations, the monomer stacking was nicely detected by UV spectroscopy by the formation of a band located at 284 nm, as previously observed at elevated pH, indicating plane to plane aggregation. However, by CD spectroscopy a bisignated signal with very low intensity was observed, indicating the formation of small aggregates with little Cotton effect amplification (see Figure S6).

A detailed inspection of the UV spectra against temperature revealed sigmoidal temperature‐dependent profiles at pH 7, in strong contrast with the previous case (Figures 3b and S7–8). The resulting data was precisely fitted with an an isodesmic or Equal‐K polymerization model (Table 2) in which a clear decrease in the degree of polymerization was observed, leading to the formation of discrete objects constituted by few monomers (see Figure S9), in good agreement with CD spectroscopy and TEM images.

**Table 2:** Thermodynamic parameters obtained from the temperature‐dependent UV/Vis, experiments of **BTT**‐**F**‐**NH_2_** ammonium formate in water at pH 7 at different concentrations using an isodesmic model. λ=300 nm.

*K*_a_^[a]^* [10^4^ M^−1^]	Δ*H* ^[b]^ [kJ mol^−1^]	Δ*S* ^[c]^ [Jmol‐1 K^−1^]	Δ*G* ^[d]^ [kJ mol^−1^]
2.9	−80	−183	−25.4

[a] Association constant. [b] Enthalpy. [c] Entropy. [d] Gibbs free energy. * K_a_ was calculated at 298 K. The cooling and heating rate were 2 K min^−1^.

In order to rule out a major influence of the ionic strength in the self‐assembly of **BTT**‐**F**‐**NH_2_** with respect to the experiments carried out at elevated pH, temperature dependent experiments at pH 7 in the absence and presence of 1 M conc of NaCl were performed. In those experiments non‐appreciable changes in the UV spectra and temperature dependent plots were appreciated (Figure S10).

Summarizing, there is a striking difference in the polymerization mechanism at the two pH values examined. The non‐protonated three amino groups exhibit a highly cooperative behaviour at pH 13, which is typically known to lead longer and monodisperse assemblies.^[18]^ However, when the terminal charged primary ammonium are formed at lower pH, the energy associated with the Coulombic interactions represents a thermodynamically unfavourable component in the overall process, being responsible for a drastic change in the polymerization mechanism, and leading to a isodesmic behavior, that can also be attributed as in previous examples to an anticooperative effect due to the frustrated growth of the aggregates.

After investigating the pH‐dependent self‐assembly of **BTT**‐**F**‐**NH_2_**, we turned our attention to the copolymerization study of mixtures of **BTT**‐**F**‐**OH** and **BTT**‐**F**‐**NH_2_** monomeric species (Figure 1a) at pH=7. Different studies of pH‐regulated supramolecular co‐polymerizations have been reported by Besenius et al., by mixing complementary charged monomeric species.^[19]^

In this case, the copolymerization will allow us to combine the fine control over structure and dispersity in a cooperative supramolecular polymer together with the frustrated growth by Coulombic repulsive forces to control the formation of columnar assemblies of defined length.

Therefore, mixtures of different ratio between co‐monomers were studied by CD and UV spectroscopy at pH=7, observing a clear reduction in the fraction of aggregated molecules when increasing the ratio of the charged monomeric species of BTT−F−NH_2_ vs. BTT−F−OH (see Figure S11).

The same trend was seen by negative staining TEM (see Figure 4), being able to gain a fine control over the length. We could go gradually from long fibers at 100 % of **BTT**‐**F**‐**OH** to very short ones in a mixture 25 : 75 of **BTT**‐**F**‐**OH** : **BTT**‐**F**‐**NH_2_**. Shortening of the fibers was supported by Zeta potential measurements (see Figure S11), going from a negative mean value for 100 % **BTT**‐**F**‐**OH** and reaching a positive maximum value at **BTT**‐**F**‐**OH**:**BTT**‐**F**‐**NH_2_** 50 : 50.

**Figure 4:**
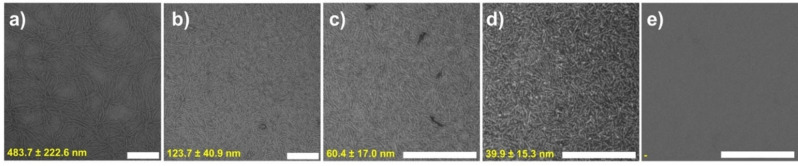
TEM images of mixtures (c=4×10^−5^ M) at pH=7 of **BTT**‐**F**‐**OH** : **BTT**‐**F**‐**NH_2_** ammonium formate at different ratios, a) 100 : 0, b) 75 : 25, c) 50 : 50, d) 25 : 75, e) 0 : 100 respectively, showing one‐dimensional fibers in water at room temperature. In yellow, average length for the fibers. Scale bar 200 nm.

In summary, the study we present unveils the delicate interplay between the forces interacting in supramolecular systems, including key mechanistic aspects of the frustrated polymerization growth induced by a combination of Coulombic repulsive forces and a higher monomer solubility in pH‐responsive processes. This mechanistic study paves the way for the preparation of size‐controlled one‐dimensional stimuli‐responsive supramolecular polymers and in aqueous media.

## Conflict of interest

The authors declare no conflict of interest.

## Supporting information

As a service to our authors and readers, this journal provides supporting information supplied by the authors. Such materials are peer reviewed and may be re‐organized for online delivery, but are not copy‐edited or typeset. Technical support issues arising from supporting information (other than missing files) should be addressed to the authors.

Supporting Information
