# Supplementary material for: Unveiling Polymerization Mechanism in pH‐regulated Supramolecular Fibers in Aqueous Media
Source: Chemistry. 2021 Jun 29;27(43):11056–60. doi: 10.1002/chem.202101660 (PMC8456867; doi:10.1002/chem.202101660)
Supplement: Supplementary file 1 — Supporting Information [file CHEM-27-11056-s001.pdf]

Chemistry–A European Journal

Supporting Information

Unveiling Polymerization Mechanism in pH-regulated Supramolecular Fibers in Aqueous Media

Nicolás M. Casellas, Lorenzo Albertazzi, Sílvia Pujals,* Tomás Torres,* and Miguel García-Iglesias*

Contents

Experimental Details S2
- Materials S2
- Methods S2
- Synthetic procedures S3

Supporting data S16
- Figures S1 and S2 .... S16
- Figures S3 and S4 .... S17
- Figures S5 and S6 .... S18
- Figures S7 and S8 .... S19
- Figures S9 and S10 .... S20
- Figures S11 and S12 .... S21

References S22

Experimental Details

Materials

Chemicals were purchased from commercial suppliers (SIGMA Aldrich and Alfa Aesar) and used without further purification unless stated otherwise. H_2N-PEG_8-OH (≥ 95%) was obtained from ChemPep Inc. All solvents were of AR quality and purchased from either Scharlau or Carlo Erba. Dry THF was degassed and obtained after passage through an activated alumina solvent column system. Water was purified on an EMD Milipore Mili-Q Integral Water Purification System. Column chromatography was carried out on silica gel (Merk, kieselgel 60, 230-400 mesh, 60 Å). Reactions were followed by thin-layer chromatography on aluminium sheets precoated 0.25 mm, 60-F254 silica gel plates from Merck. All reactions were performed under an atmosphere of dry argon unless stated otherwise. **BTT-F-OH** derivative was synthetized according to the procedure previously described by us.[1]

Methods

^{1}H-NMR and ^{13}C-NMR spectra were recorded on a Bruker AC-300 or a Bruker AC-500 spectrometer. Chemicals shifts are given in ppm (δ) values relative to residual solvent or tetramethylsilane (TMS). Splitting patterns are labeled as s, singlet; d, doublet; dd, double doublet; t, triplet; q, quartet; quin, quintet; m, multiplet and b stands for broad. MS (MALDI-TOF) spectra were performed on a BRUKER REFLEX III instrument that was equipped with a nitrogen laser operating at 337 nm and recorded in the positive-polarity mode. High-resolution spectra were acquired using a 9.4 T IonSpec QFT-MS FT-ICR mass spectrometer. Some samples were analyzed in a mass spectrometer with hybrid analyzer QTOF model MAXIS II of the commercial house Bruker. An Acquity UPLC from the commercial house Waters was used as an entryway in Flow Injection analysis mode (FIA).

Ultraviolet-visible (UV-vis) absorbance spectra and Fluorescence spectra were recorded on a Jasco V-660-spectrophotometer and a Jasco FP-8600 spectrofluorometer respectively, both of them with a Jasco Peltier ETCS-761 temperature controller incorporated. Circular dichroism (CD) spectra were recorded on a Jasco J-815 CD-spectrometer including a Jasco Peltier ETCT-761 temperature controller.

UV-vis, fluorescence and CD spectroscopy measurements were performed using quartz cuvettes (1cm). Solutions were prepared by weighting in the necessary amount of compound for a given concentration. Water solutions were prepared by injecting a concentrated DMSO solution (1 x10^{-2} M) into water mili-Q to obtain the desired final concentration. In all cases, the solutions were optically transparent.

For Transmission Electron Microscopy (TEM) samples were deposited onto C-only grids and negative staining was performed using uranyl acetate at 2%. All electron micrographs were obtained with a Jeol JEM 1010 MT electron microscope (Japan) operating at 80 kV. Images were obtained on a CCD camera Megaview III (ISIS), Münster, Germany.

pH values were recorded using a Hanna pH tester HI98103, with an electrode HI1271. The resolution is 0.1 pH and and the accuaracy ± 0.2 pH.

Synthetic procedures

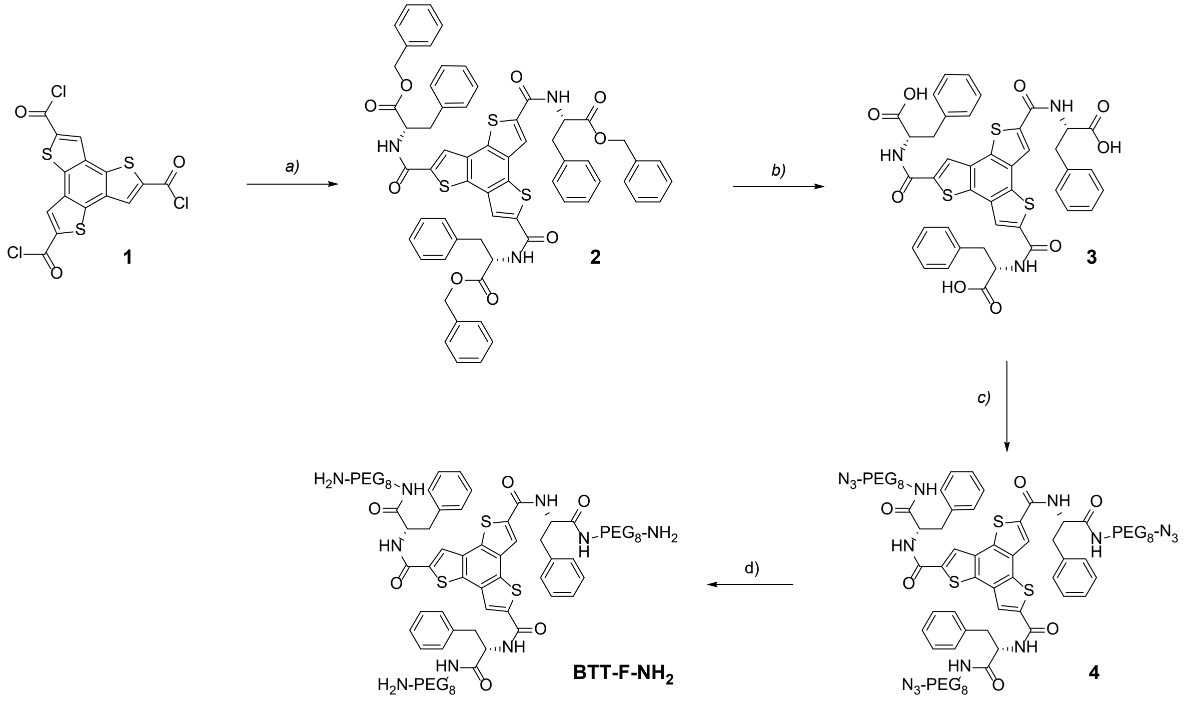

Scheme 1: *a) L*-Phenylalanine benzyl ester hydrochloride*,* Et3N, THF, reflux, 15 h, 52%. *b)* H_2, Pd/C, MeOH, R.T, 12 h, 30%. c) H_2N-PEG_8-N_3, DMTMM, THF, R.T, 12h, 76%. d) PPh_3, H_2O, THF, R.T, 24h, 73%.

BTT-*L*-Phenylalanine benzyl ester (2)

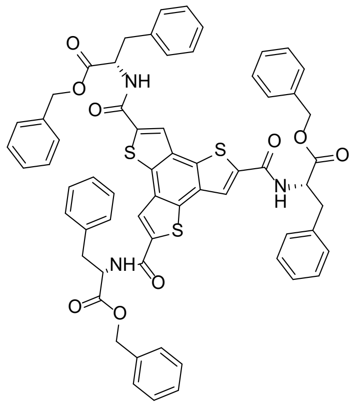

To a suspension of compound **1**[1] (0,2 g, 0,46 mmol) in dry THF (74 mL) was added *L*-Phenylalanine benzyl ester hydrochloride (0,67 g, 2,29 mmol) and triethyl amine (0,32 mL, 2,29 mmol). The mixture was refluxed for 15 h and the solvent was removed in vacuum. The product was purified by column chromatography in silica gel in $CHCl_3$ (2% MeOH) to obtain **2** as a pale yellow solid 0,26 g (52%).

^{1}H NMR (300 MHz, DMSO-d_6) δ 9.37 (d, J = 6.8 Hz, 3H, NH), 8.61 (s, 3H, core), 7.42 – 7.11 (m, 30H, ArH), 5.29 – 5.02 (dd, J= 3.1 Hz, 12,8 Hz, 6H, <u>CH_2</u>Bn), 4.87 – 4.66 (dd, J= 7.8 Hz, 9.1 Hz, 3H, <u>CH</u>-$COOCH_2$Ar), 3.29 – 3.11 (m, 6H, <u>CH_2</u>Ar).^{13}C NMR (101 MHz, $CDCl_3$-d, δ) 171.85, 161.52, 138.40, 136.35, 135.88, 135.40, 131.45, 129.89, 129.23, 129.19, 127.86, 123.59, 68.13, 54.24, 38.34. FT-IR (ATR) ν (cm^{-1}): 3218, 3024, 2320, 2209, 2111, 1729, 1628, 1541, 1495, 1453, 1361, 1333, 1270, 1238, 1207, 1164, 1123, 1061, 965, 904, 841, 796, 746, 693. MS (MALDI; DCTB + PPGNa 1000 + NaI): m/z calc $C_{63}H_{51}N_3O_9S_3$: 1112.2692 (100) $[M+Na]^+$; found 1112.2689 (100) $[M+Na]^+$.

^{1}H NMR of 2 (DMSO-d_6)

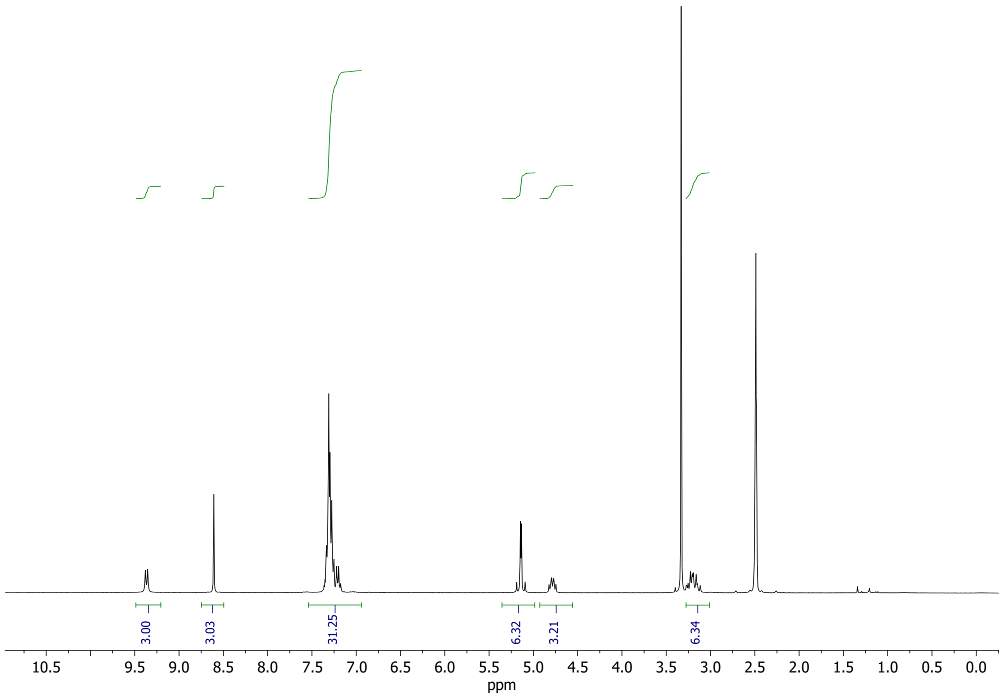

^{13}C NMR of 2 (CDCl$_3$-*d*)

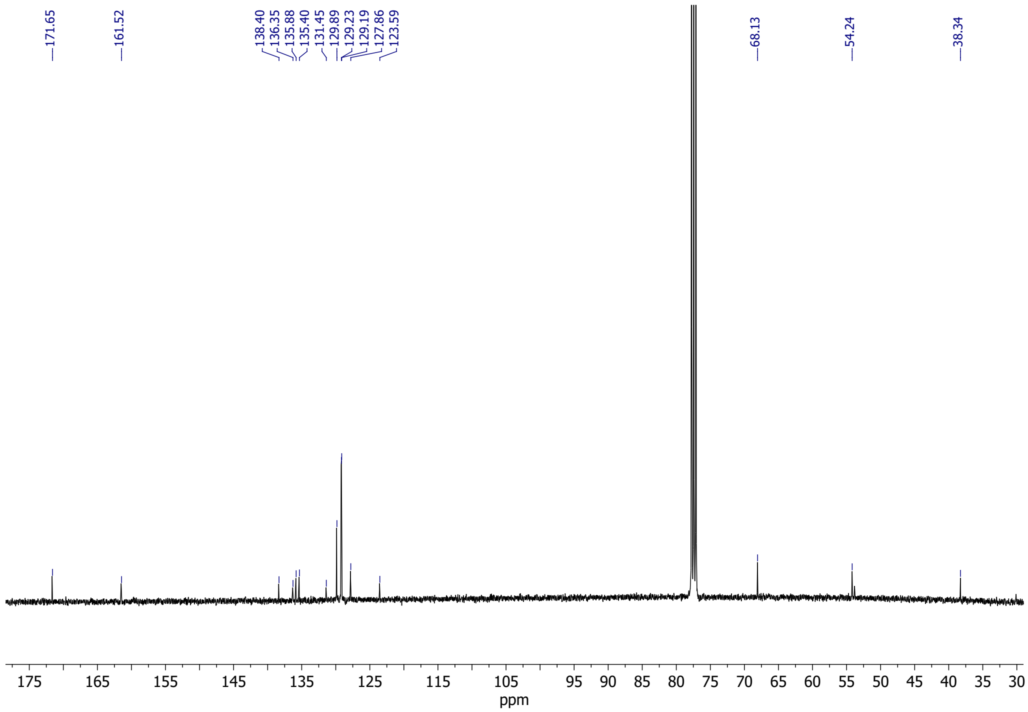

MALDI-TOF spectra with (inset) isotopic distribution HR-MALDI-TOF spectrum of 2

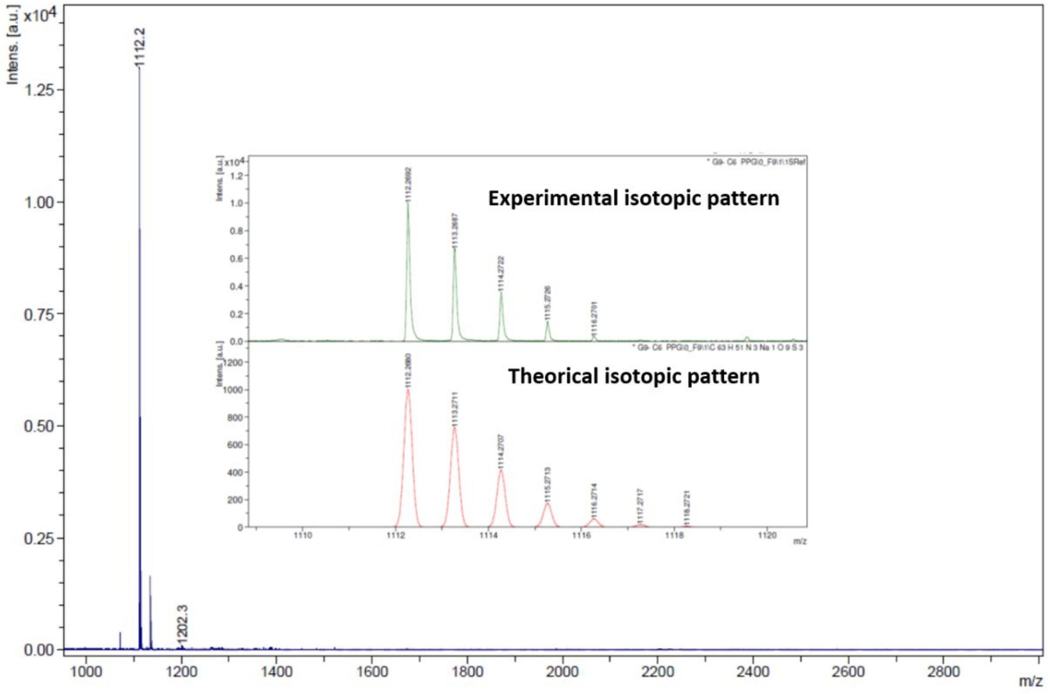

BTT-*L*-Phenylalanine (3)

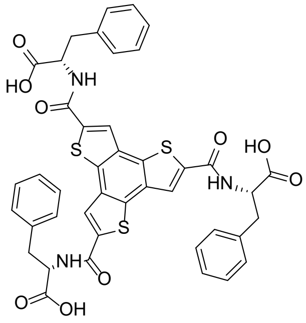

A round bottom flask (100 mL) was charged with **2** (0.2 g, 0.183 mmol) and methanol (40 mL) and the solution was purged with argon. Then, a catalytic amount of Pd/C was added and a balloon filled with H_2 (g) was connected. The reaction mixture was stirred under H2 (g) atmosphere overnight at room temperature. Subsequently, the black powder was filtered over celite® and concentrated in vacuo yielding **3**, 0,05 g (30%) as a white solid.

^{1}H NMR (300 MHz, DMSO-*d*$_6$,) δ (ppm) 9.03 (d, *J* = 7.4 Hz, 3H, NH), 8.63 (s, 3H, core), 7.42 – 7.14 (m, 5H, ArH), 4.67 (m, 3H, CH), 3.09 (dd, *J* = 13.9, 10.3 Hz, 6H, CH_2Ar). ^{13}C NMR (100 MHz, DMSO-*d*$_6$) δ (ppm) 160.70, 139.63, 138.08, 134.85, 130.98, 129.06, 128.37, 128.23, 127.60, 126.37, 123.47, 54.69,

36,67. FT-IR (ATR) ν (cm^{-1}): 3466.027, 3288.064, 2869.541, 1737.165, 1645.702, 1539.643, 1455.192, 1358.084, 1284.628, 1248.349, 1102.160, 947.432, 842.999, 701.280. MS (FB^+; m-NBA): m/z calc $C_{42}H_{33}N_3O_9S_3$: 819.14 (100) $[M+H]^+$; found 820.10 (60) $[M+H]^+$, 655.0 (100)$[M-C_9H_{10}NO_2]^+$.

^{1}H NMR of 3 (DMSO-d_6)

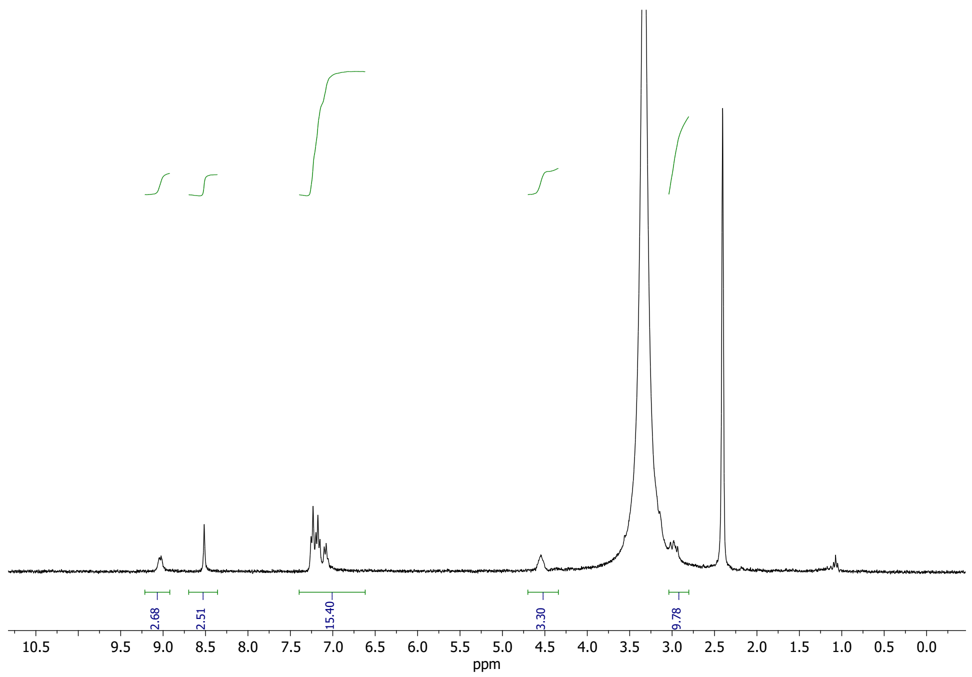

^{13}C NMR of 3 (DMSO-d_6)

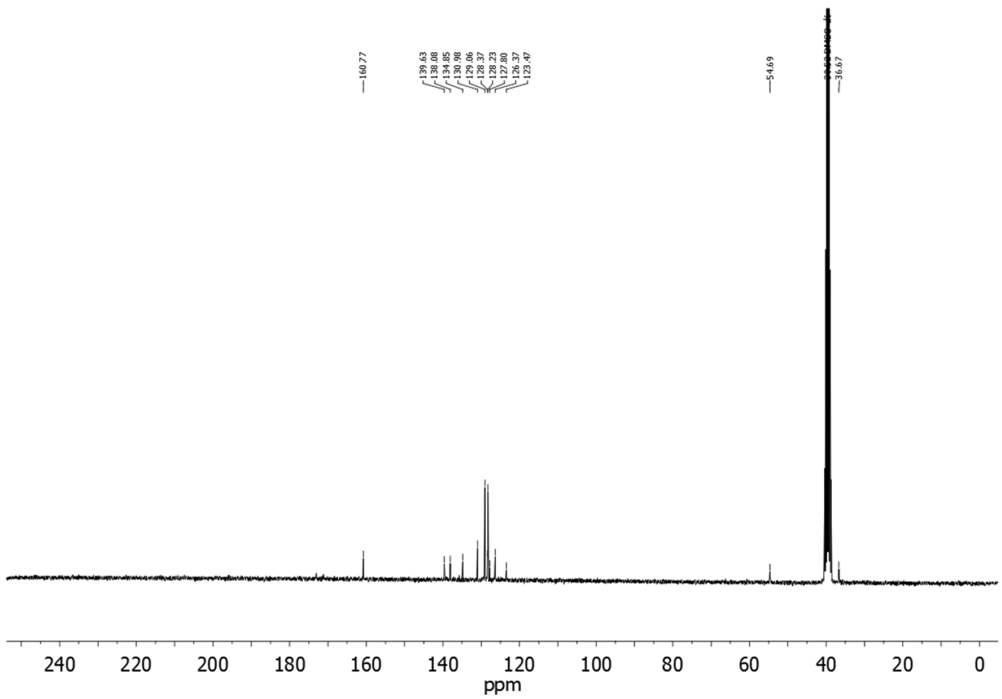

FAB spectra with (inset) isotopic distribution pattern spectrum of 3

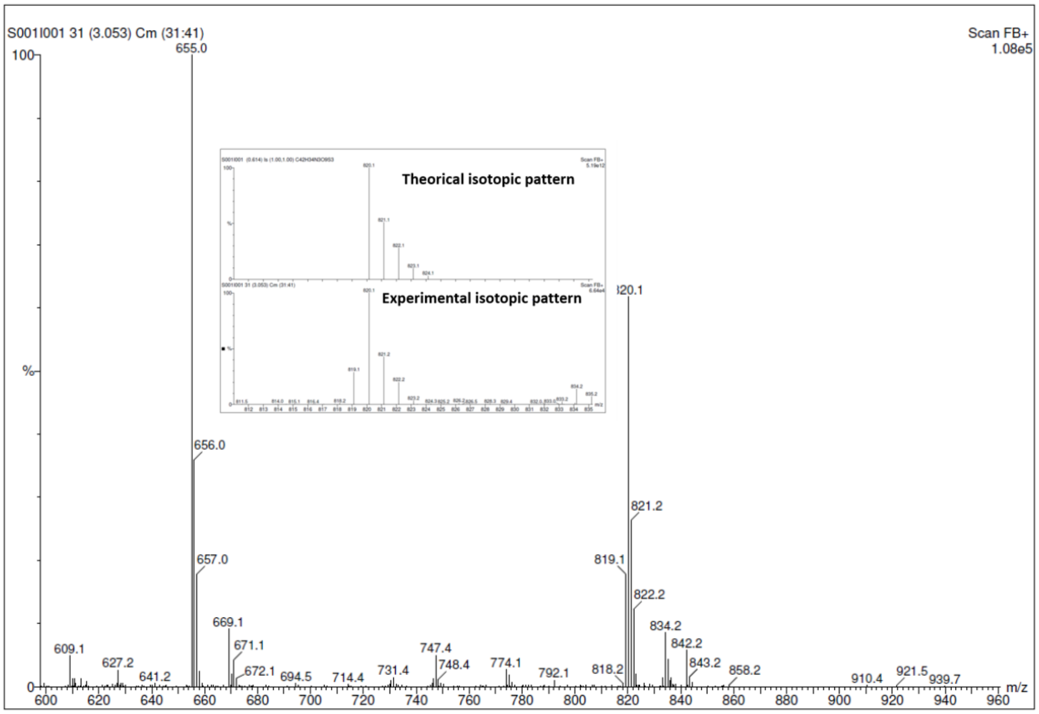

BTT-4

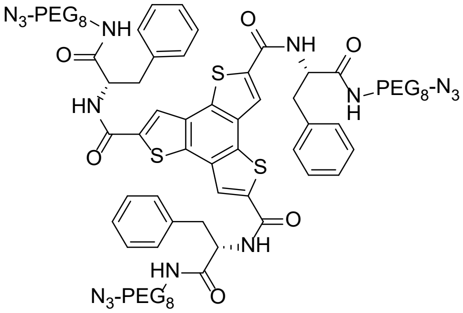

In a round bottom flask H_2N-PEG_8-N_3 (0.07 g, 0.15 mmol) is added to a mixture of compound **3** (0.1 g, 0.12 mmol), DMTMM (0.05 g, 0.18 mmol) in dry THF (25 mL). Subsequently, the reaction mixture was stirred overnight under Argon atmosphere. The mixture was concentrated *in vacuo* and it was purified by column chromatography in silica gel with a mixture of chloroform:methanol (95/5 v/v) as eluent to afford **4** as a colorless oil (0.19 g, 0.09 mmol, 76%).

^{1}H NMR (300 MHz, $CDCl_3$, δ) 7.95 (s, 3H, core) 7.81 (br, 3H, NH), 7.32-7.28 (m, 15H, ArH), 7.08 (s, 3H, NH), 4.93 (dd, J = 7.1 Hz, 14.3 Hz, 3H, CH), 3.71 – 3.46 (m, 102H, PEG), 3.25 (d, *J* = 7.2 Hz, 6H, CH_2Ar).

^{13}C NMR (101 MHz, $CDCl_3$, δ) 168.16, 161.72, 138.23, 137.11, 135.55, 130.81, 129.50, 128.67, 127.00, 123.16, 77.16, 70.77, 70.74, 70.71, 70.68, 70.65, 70.61, 70.47, 70.29, 70.10, 70.08, 69.60, 55.61, 54.64, 54.60, 53.54, 50.75, 40.90, 39.62. FT-IR (ATR) ν (cm^{-1}): 3289, 2915, 2870, 2109 (N_3), 1683, 1600, 1565, 1546, 1468, 1458, 1372, 1332, 1245, 1198, 1116, 1090, 1037, 959, 920. MS (MALDI-TOF, DCTB + NaI: *m/z* (%):$C_{96}H_{141}N_{15}O_{30}S_3$: 2104.8 (100) $[M+Na]^+$. HR MALDI-TOF MS, DCTB + PPGNa 2000 + NaI: m/z calc $C_{96}H_{141}N_{15}O_{30}S_3$: 2103.9023 (100) [M+Na]; found 2103.8954 (100) [M+Na].

^{1}H NMR of 4 ($CDCl_3$)

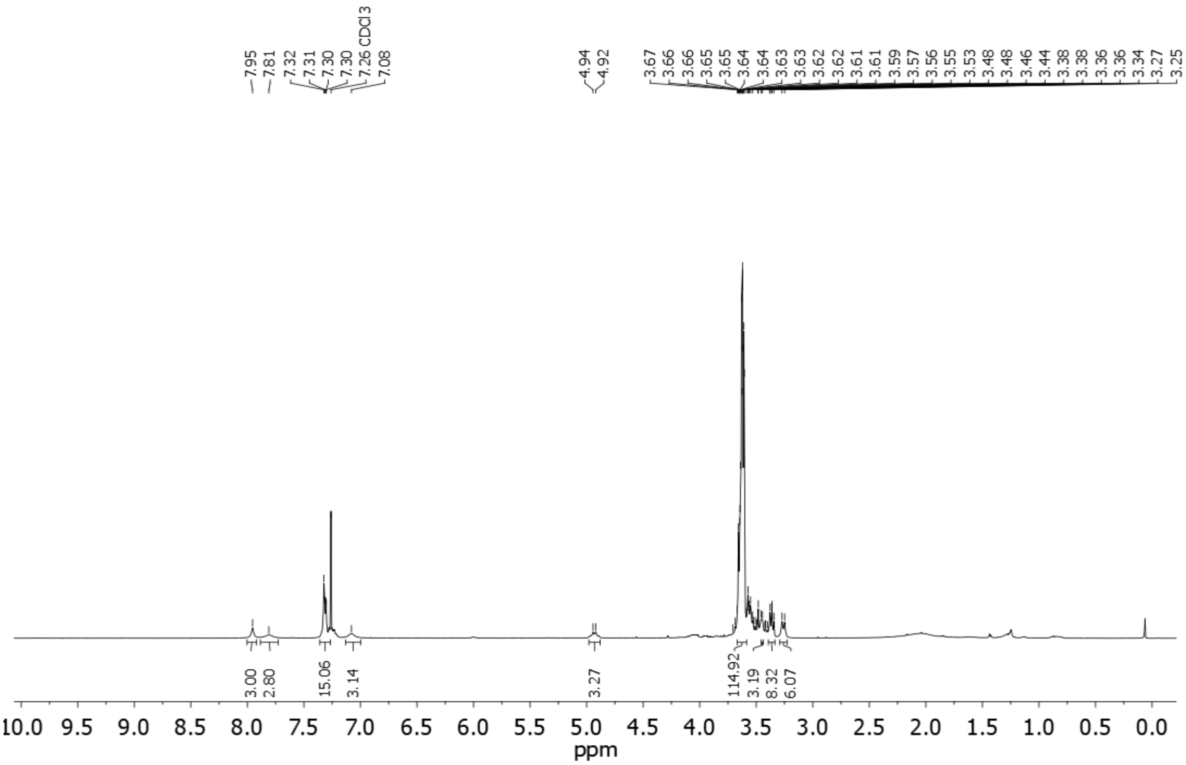

^{13}C NMR of 4 ($CDCl_3$)

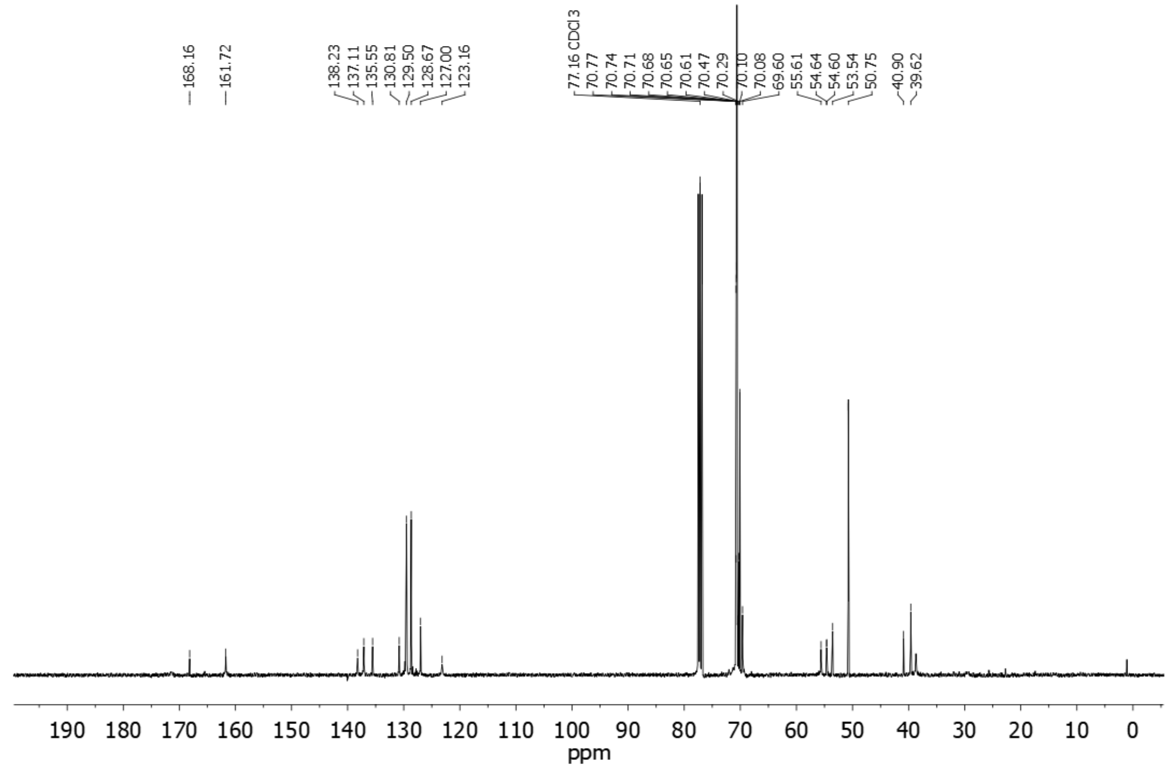

MALDI-TOF spectra with (inset) isotopic distribution pattern HR-MALDI-TOF spectrum of 4.

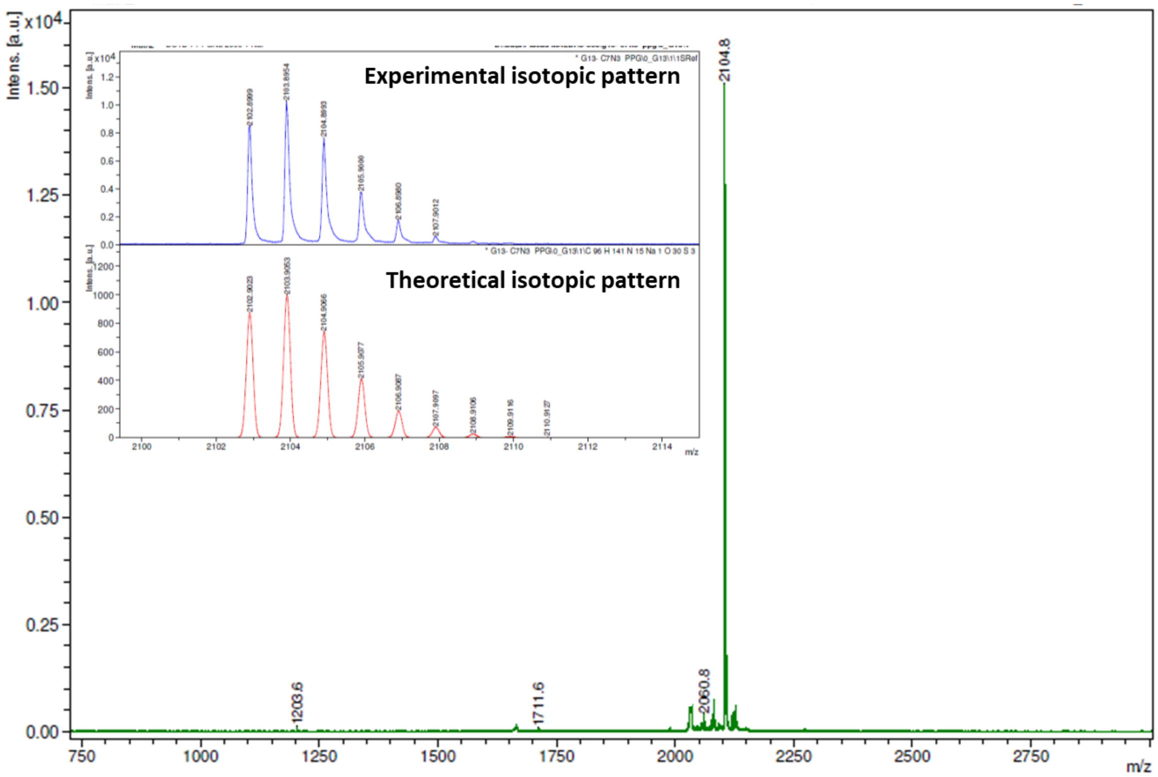

FT-IR (ATR) of 4

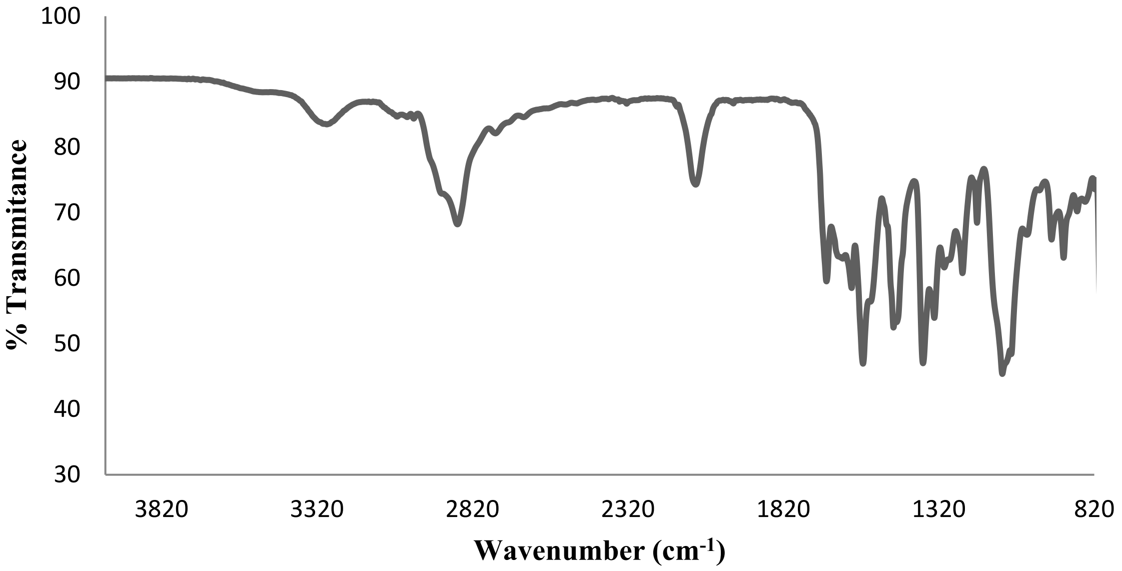

BTT-F-NH$_2$

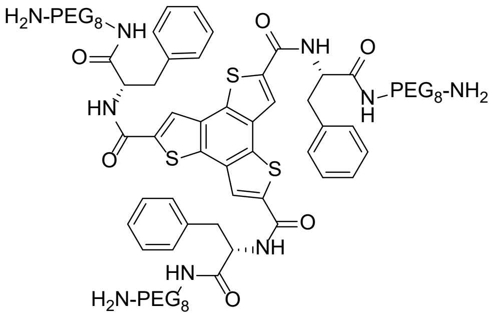

A solution of **4** (0.10 g, 0.048 mmol) in THF (4.0 mL) was added water (6 µL, 0.29 mmol) at room temperature for 5 minutes. Then, triphenylphosphine (0.08 g, 0.29 mmol) was added to the reaction mixture and it was stirred for 24 h at room temperature. The solvent was removed in vacuum and purified by column chromatography in deactivated silica gel into a mixture of chloroform:methanol:ammonia (89/10/1 v/v). The organic extract was washed with water (1% $NaHCO_3$) and dried over anhydrous $MgSO_4$. Purification was performed with a Waters semipreparative-HPLC and characterization with a HPLC Waters e2695 separation module equipped with Photodiode Array detector Waters 2998 and Mass spectrometer QDA detector Acquity. A XSelect CSH C18 3.5µm 4.6x50mm was used. Water 0.5% HCOOH and ACN 0.5% HCOOH were used as eluents. Subsequently, the solvent was removed to afford **BTT-F-NH$_3^+$COO$^-$** as a yellowish oil in a yield of 68% (0.07 g, 0.035 mmol).

^{1}H NMR (300 MHz, $CDCl_3$, δ) 8.20 (br, 3H, NH), 7.85 (s, 3H, core), 7.59 (br, 3H, NH), 7.35-7.21 (m, 15H, Ar) 4.96 (d, *J* = 7.9 Hz, 3H, CH), 3.61 – 3.53 (m, 102H, PEG), 2.85 (m, 12H, CH_2-NH_6 and NH_2). ^{13}C NMR (101 MHz, $CDCl_3$, δ) 171.66, 162.63, 161.89, 138.33, 137.86, 137.29, 136.12, 135.64, 133.26, 130.83, 130.35, 129.61, 128.65, 127.30, 126.94, 125.27, 123.26, 72.83, 70.65, 70.59, 70.57, 70.55, 70.54, 70.32, 70.28, 69.73, 61.71, 55.64, 39.65, 38.58. FT-IR (ATR) ν (cm^{-1}): 3319 (NH_2), 3058, 2935, 2871, 1648, 1587, 1542, 1478, 1437, 1361, 1298, 1251, 1183, 1118, 1092, 1069, 1029, 951, 915. MS (MALDI-TOF, DCTB + NaI): *m/z* (%):$C_{96}H_{147}N_9O_{30}S_3$: 2025.8 (100) [M+Na]$^+$. HR MALDI-TOF MS, DCTB + PPGNa 2000 + NaI): m/z calc $C_{96}H_{147}N_9O_{30}S_3$: 2024.9330 (100) [M+Na]$^+$; found: 2024.9308 (100) [M+Na]$^+$.

HPLC of BTT-F-NH_2

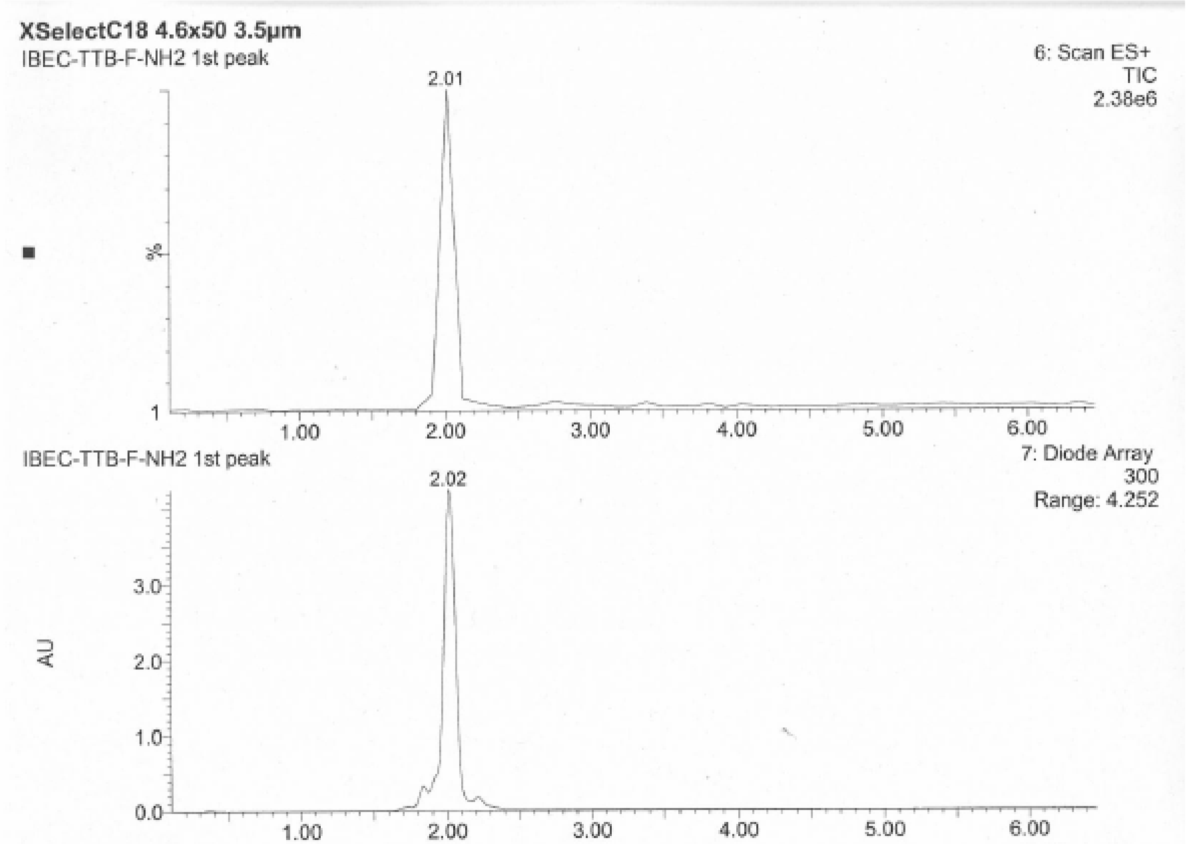

^{1}H NMR of BTT-F-NH$_2$ (CDCl$_3$)

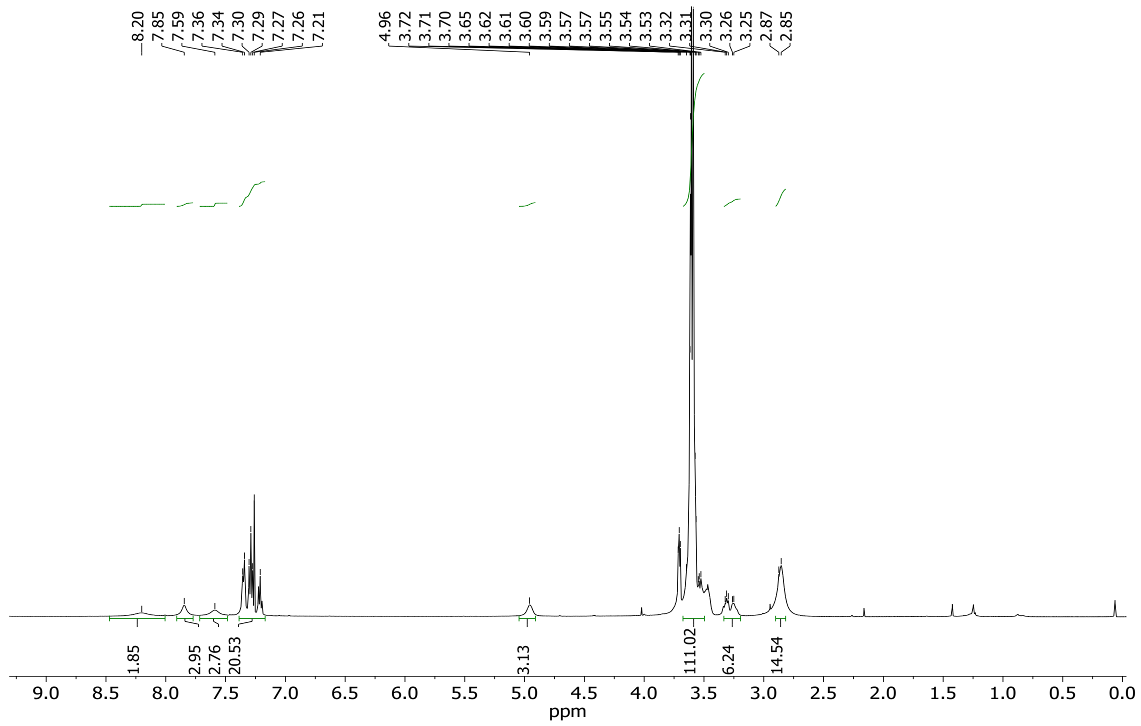

^{13}C NMR of BTT-F-NH$_2$ (CDCl$_3$)

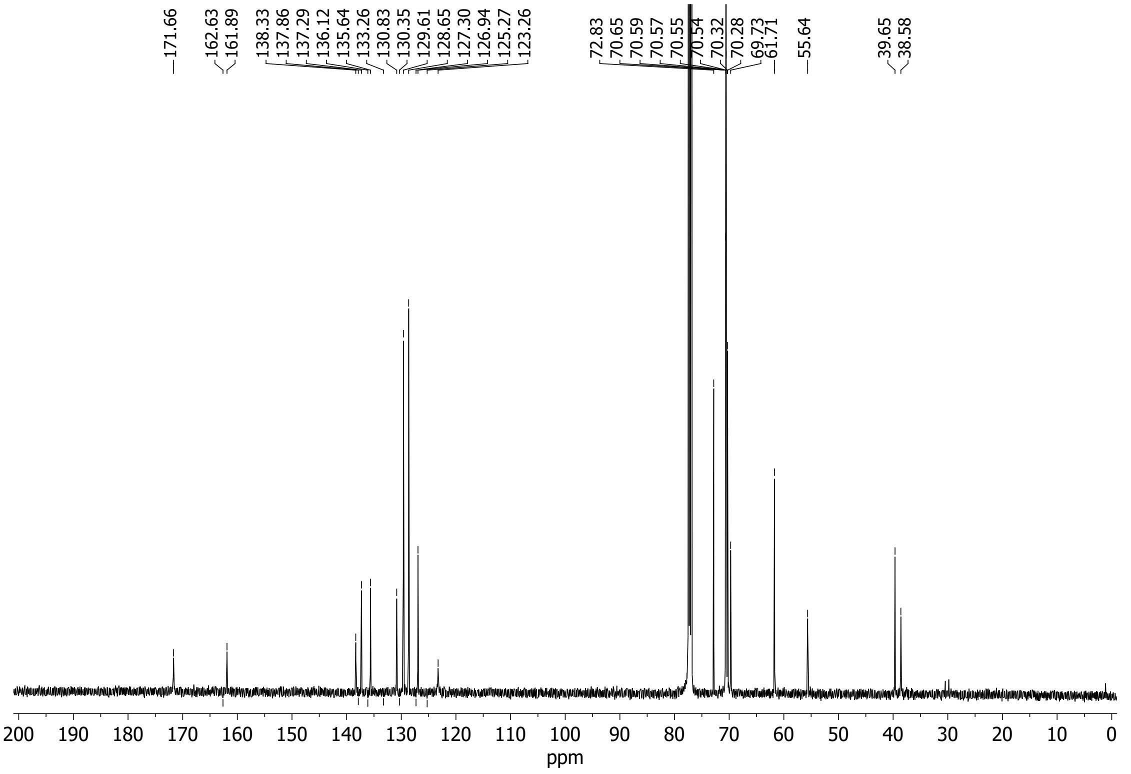

MALDI-TOF spectra with (inset) isotopic distribution pattern HR-MALDI-TOF spectrum of BTT-F-NH_2.

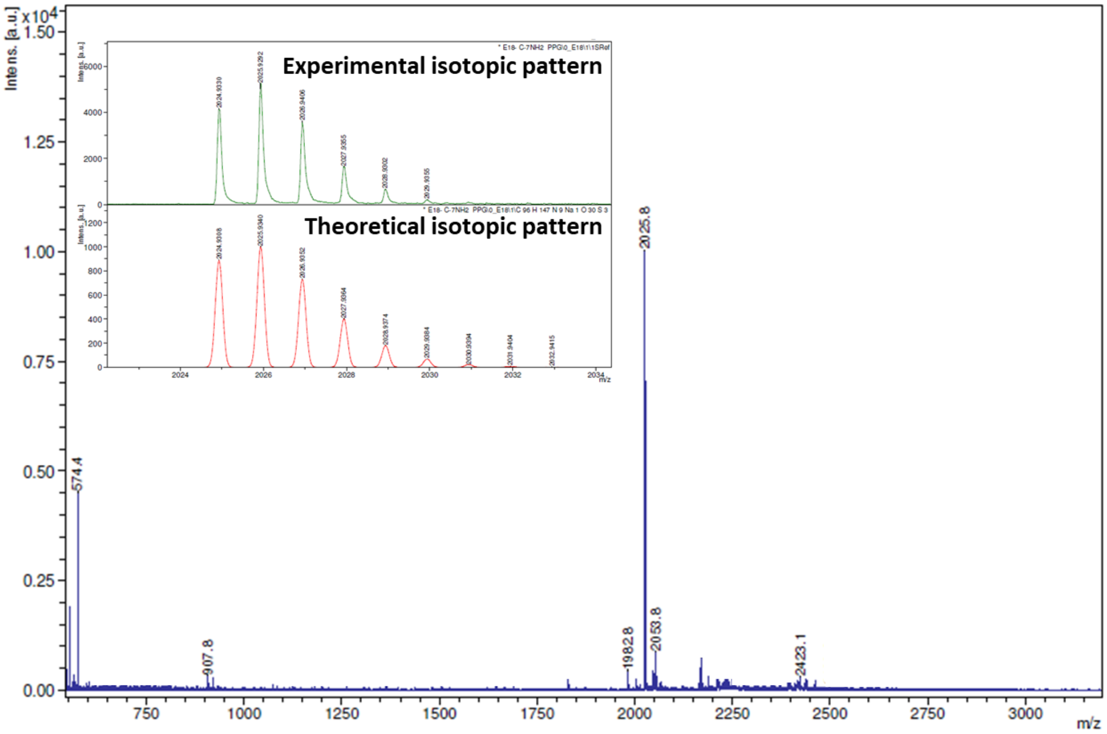

FT-IR (ATR) of BTT-F-NH_2

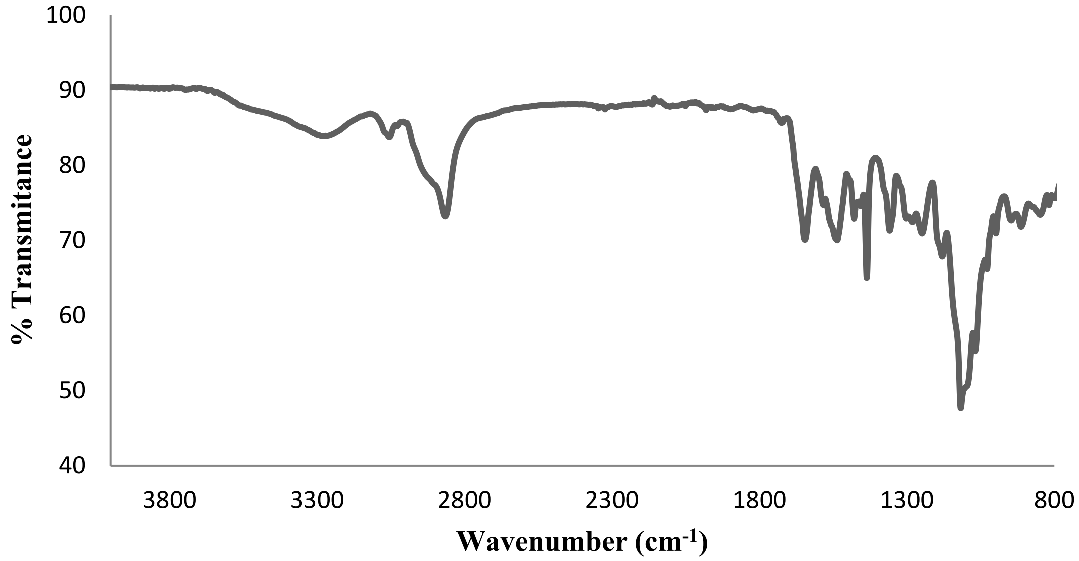

Supporting data

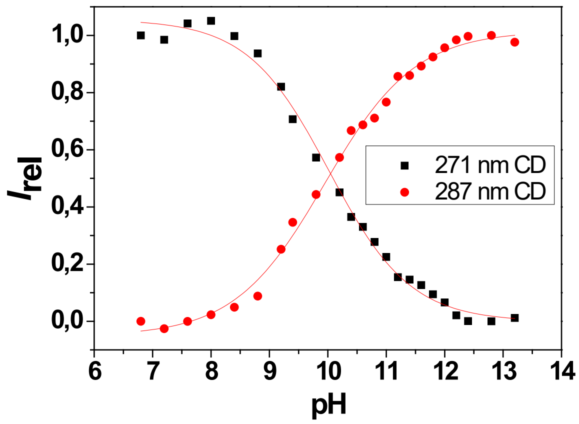

$I = I_{min} + (I_{max} - I_{min}) / (1 + 10^{(pH - pKa)})$

CD Fitting	*Parameters*
**271 nm**	I_{min} = -0,001
	I_{max} = 1,054
	**pKa =** 10,017
	R-square = 0,9946
**287 nm**	I_{min} = -0,02
	I_{max} = 1,016
	**pKa =** 9,94
	**R-square =** 0,9953

Figure S1: Normalized CD signal intensity I_{rel} at λ = 287 nm (red dots) and λ = 271 nm (black dots) of **BTT-F-NH$_2$** ammonium formate (c = 5 x 10^{-5} M) fitted with the non-protonated/protonated ratio using the Henderson−Hasselbalch equation (line) $I = I_{min} + (I_{max} - I_{min}) / (1 + 10^{(pH - pKa)})$ as a function of pH (line).

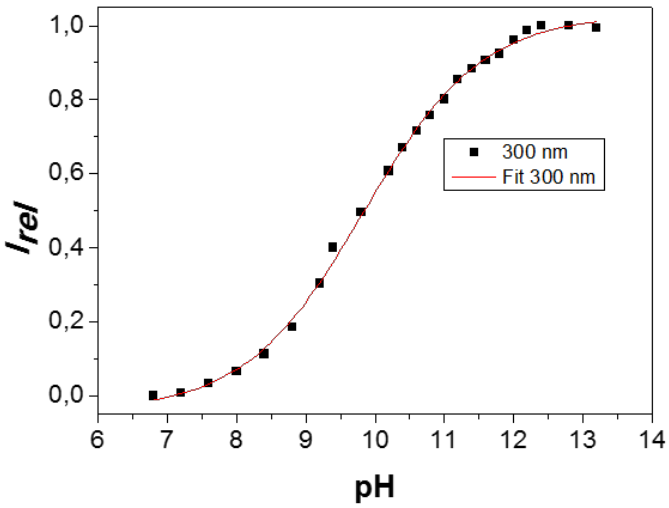

$I = I_{min} + (I_{max} - I_{min}) / (1 + 10^{(pH - pKa)})$

UV Fitting	*Parameters*
**300 nm**	I_{min} = -0,04
	I_{max} = 1,03
	**pKa =** 9,89
	R-square = 0,9985

Figure S2: Normalized UV signal intensity I_{rel} at λ = 287 nm (red dots) and λ = 271 nm (black dots) of **BTT-F-NH$_2$** ammonium formate (c = 5 x 10^{-5} M) fitted with the non-protonated/protonated ratio using the Henderson−Hasselbalch equation (line) $I = I_{min} + (I_{max} - I_{min}) / (1 + 10^{(pH - pKa)})$ as a function of pH (line).

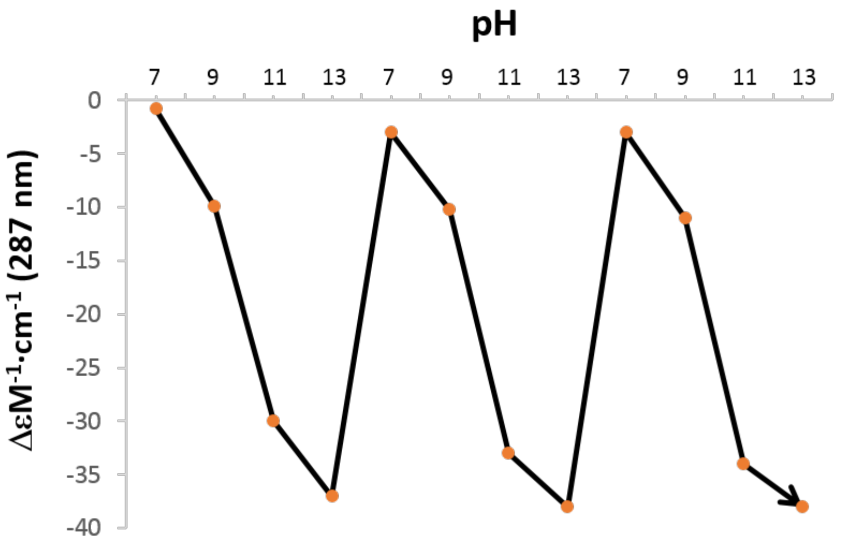

Figure S3: Reversibly switched CD of **BTT-F-NH₂** (c = 5 x 10^{-5} M) by repeated pH cycling adjusted by addition of NaOH and HCl.

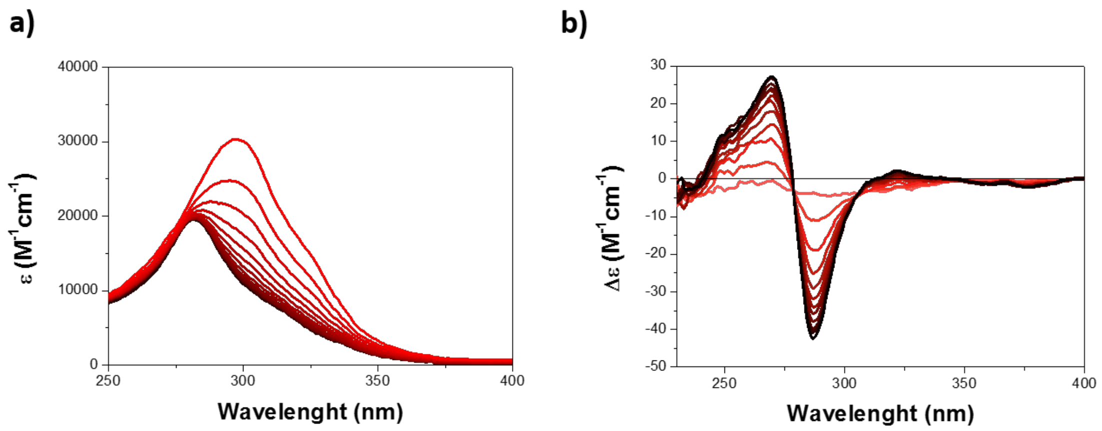

Figure S4: Temperature dependent spectra of **BTT-F-NH₂** in water (c= 7.3 x 10^{-6} M) determined by UV (a) and CD (b) spectroscopy at pH = 13. The heating direction from 273 K to 353 K is indicated from black to red every 10 degree.

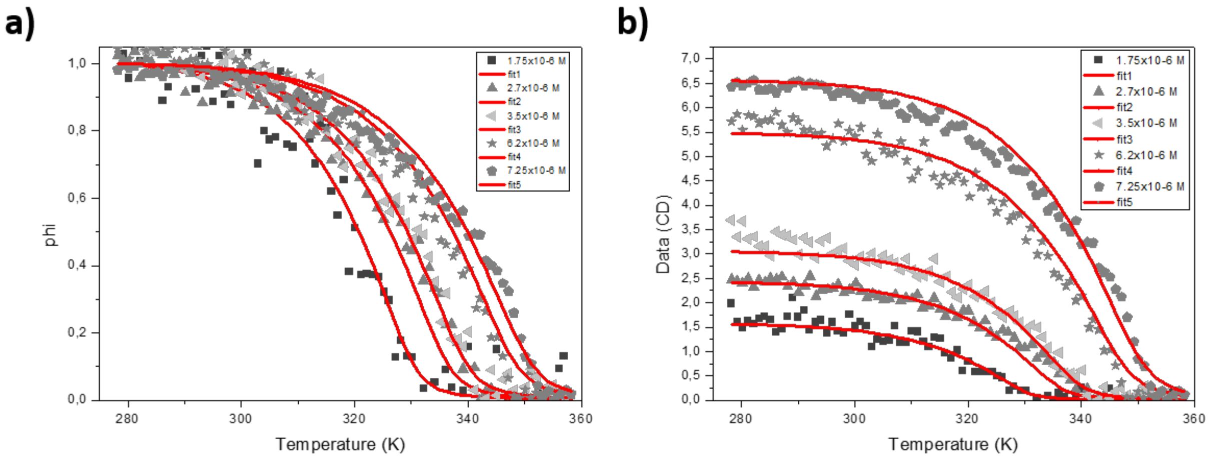

Figure S5: Fit of the normalized cooling curves in water (a) degree of aggregation phi and (b) data of **BTT-F-NH$_2$** determined by temperature dependent CD (λ = 287 nm), at different concentrations at pH = 13. Thermodynamic parameters derived from the fit using a cooperative model: ΔH_{ELO} = -65 kJ mol^{-1}, ΔS = -85 Jmol^{-1}K^{-1}, ΔH_{NP} = -26 kJ mol^{-1}, K_e = 9.1 x 10^6 M^{-1}, K_n = 214 M^{-1}, σ = 2.3 x 10^{-5}. T_e = 349, 346, 338, 334, 329 K. K_n, K_e and σ were calculated at 298 K. The cooling and heating rate were 2 K min^{-1}.

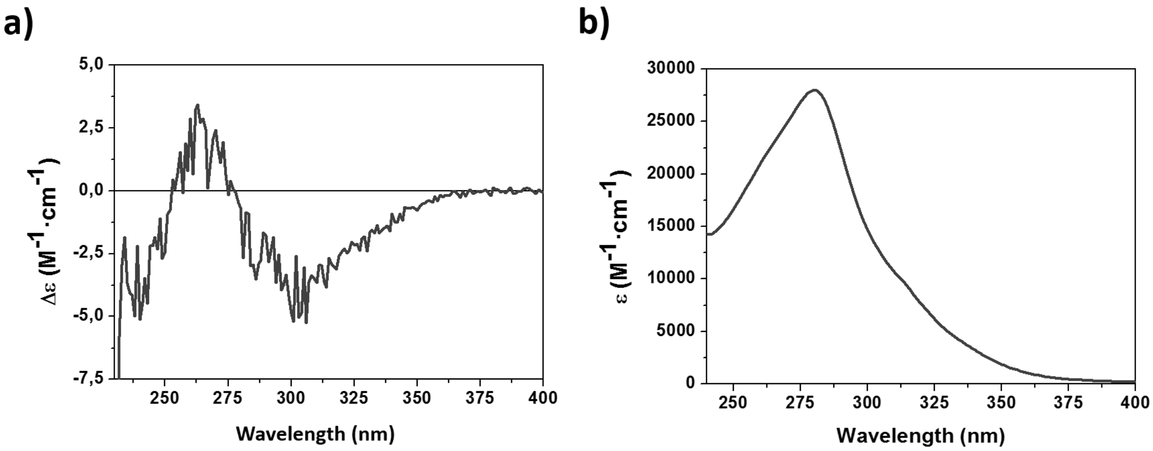

Figure S6: BTT-F-NH$_2$ spectra in water (c= 7.3 x 10^{-6} M) at pH = 7 determined by UV (a) and CD (b) spectroscopy.

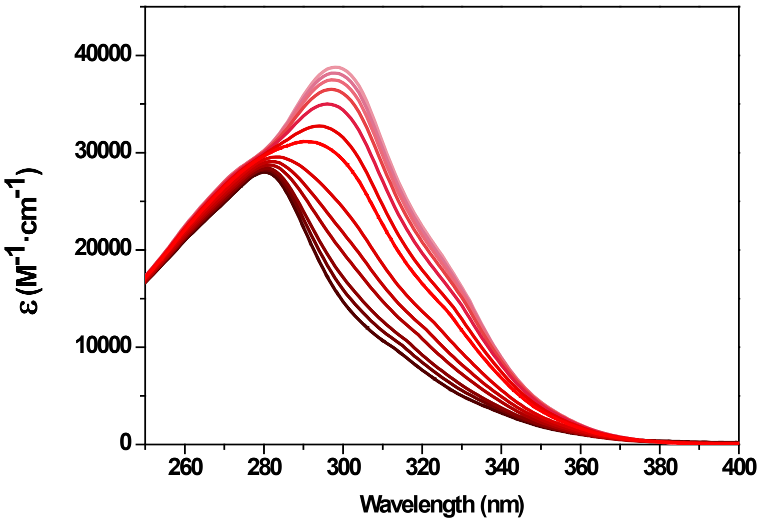

Figure S7: Temperature dependent spectra of **BTT-F-NH$_2$** in water at pH = 7 (c= 1 x 10^{-4} M) determined by UV spectroscopy. The heating direction from 273 K to 353 K is indicated from black to red every 10 degree.

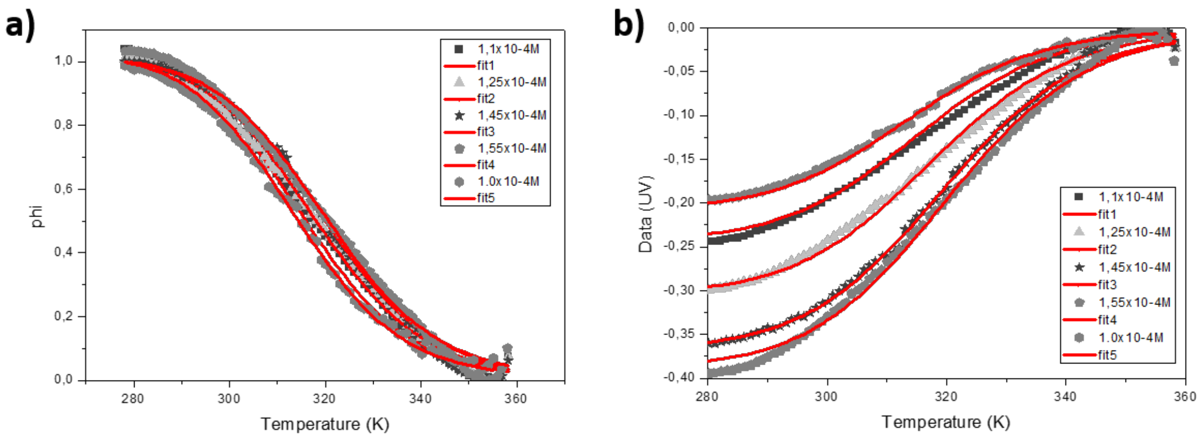

Figure S8: Fit of the cooling curves in water (a) degree of aggregation phi and (b) UV variation (λ = 300 nm) of **BTT-F-NH$_2$** ammonium formate determined at different concentrations at pH = 7. Thermodynamic parameters derived from the fit using an isodesmic model: $K_{a\,=}$ 2.9 x 10^4 M^{-1}, ΔH = -80 kJ mol^{-1}, ΔS = -183 J mol^{-1} K^{-1}, and ΔG = -25.4 kJ mol^{-1}, T_m = 315, 314, 312, 310, 308 K. K_a was calculated at 298 K. The cooling rate was 2 K min^{-1}.

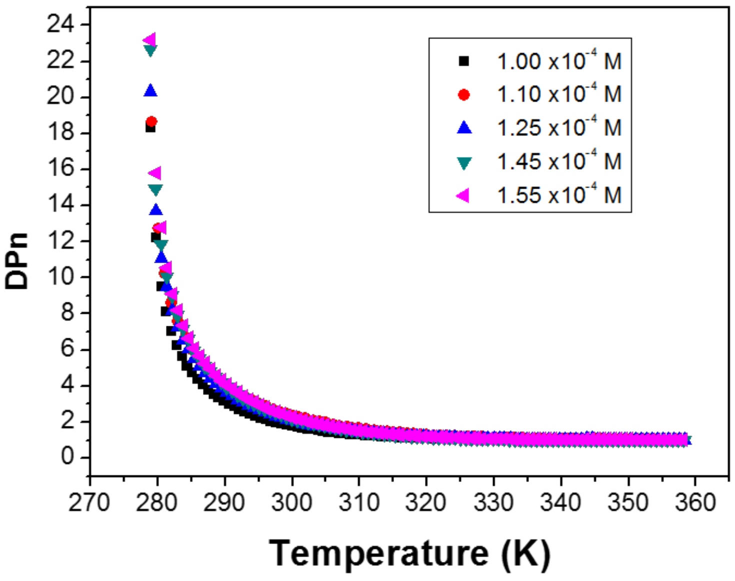

Figure S9: Number-averaged degree of polymerization (DPN) $\left(DPn = \frac{1}{\sqrt{1-\alpha agg\ (T)}}\right)^2$ of **BTT-F-NH$_2$** at different temperatures and concentrations. DPn 1.00 x 10^{-4} (298 K) = 1,61, DPn 1.25 x 10^{-4} (298 K) = 1,73, DPn 1.45 x 10^{-4} (298 K) = 2,00, DPn 1.55 x 10^{-4} (298 K) = 2,16.

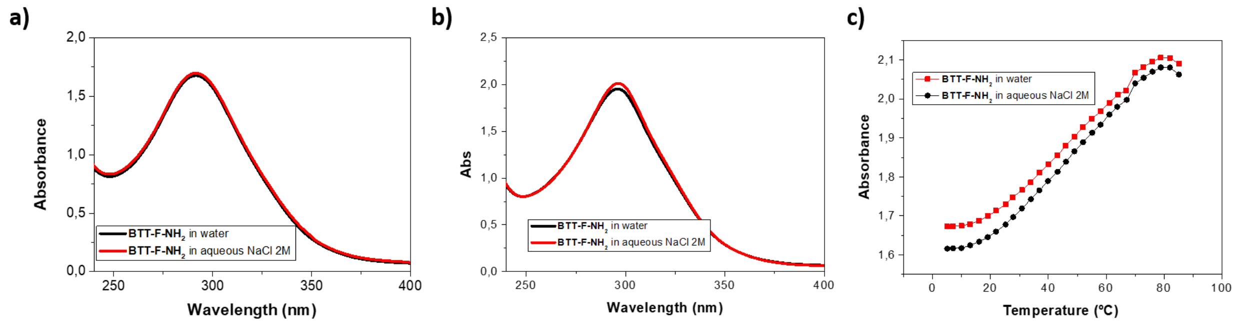

Figure S10: UV Temperature dependent experiments of **BTT-F-NH$_2$** ammonium formate in water at pH = 7 (c= 1.8 x 10^{-4} M) performed in the absence (black) and presence (red) of 2M NaCl concentration. (a) UV spectra recorded at 10 ºC; (b) UV spectra recorded at 75ºC. (c) Plot of the UV intensity over temperature by using a cooling rate of 2 K min^{-1}.

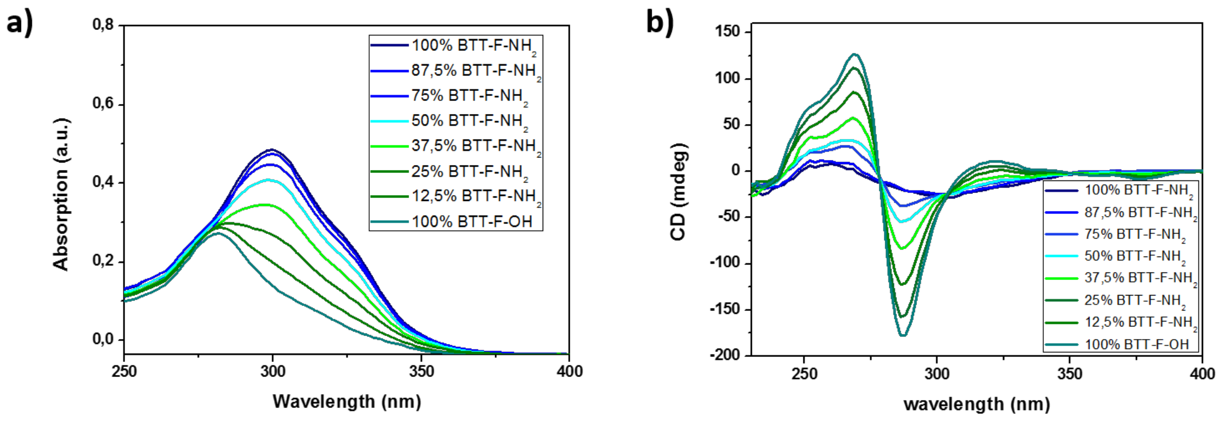

Figure S11: Mixtures at different ratios at pH 7 of **BTT-F-NH$_2$** ammonium formate **and BTT-F-OH** keeping the monomer concentration constant (1.6 x 10^{-5} M) (a) UV and (b) CD spectroscopy.

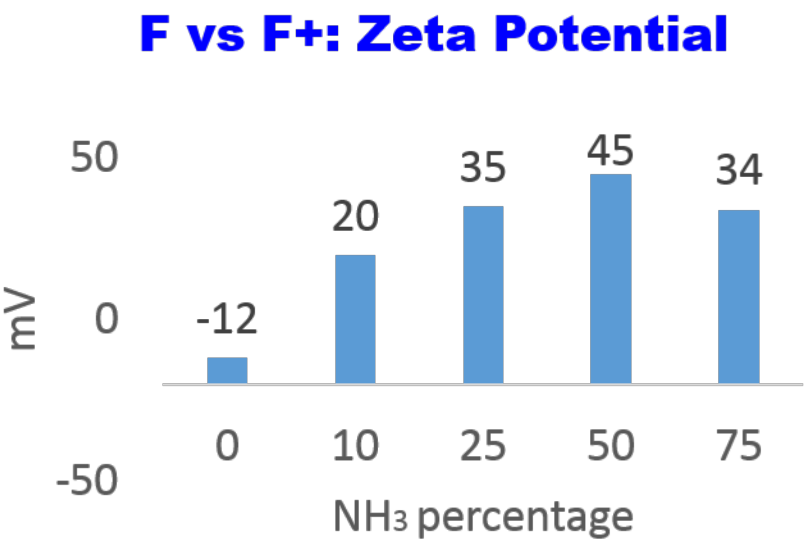

Figure S12: Zeta potential mean value depending on the percentage of **BTT-F-NH$_2$**, in mixtures **BTT-F-NH$_2$** ammonium formate /**BTT-F-OH**.

References

1 N. M. Casellas, S. Pujals, D. Bochicchio, G. M. Pavan, T. Torres, L. Albertazzi, M. García-Iglesias, *Chem. Comm.* **2018**, *54*, 4112.

2 (a) A. Demenev, S. H. Eichhorn, *Chem. Mater.* **2010**, 22, 1420–1428; (b) X. Guo, S. Wang, V. Enkelmann, M. Baumgarten K. Müllen, *Org. Lett.* **2011**, *13*, 6062.

3 N. K. Allampally, A. Florian, M. J. Mayoral, C. Rest, V. Stepanenko, G. Fernández, *Chem. - Eur. J.* **2014**, *20*, 10669.
